# Does Acupuncture Protect Dopamine Neurons in Parkinson's Disease Rodent Model?: A Systematic Review and Meta-Analysis

**DOI:** 10.3389/fnagi.2019.00102

**Published:** 2019-05-08

**Authors:** Jade Heejae Ko, Hyangsook Lee, Seung-Nam Kim, Hi-Joon Park

**Affiliations:** ^1^College of Korean Medicine, Dongguk University, Goyang, South Korea; ^2^Graduate School, Dongguk University, Seoul, South Korea; ^3^Acupuncture and Meridian Science Research Center, Seoul, South Korea; ^4^College of Korean Medicine, Kyung Hee University, Seoul, South Korea

**Keywords:** acupuncture, dopamine neuroprotection, meta analysis, Parkinson's disease, rodent model, systematic review

## Abstract

**Background:** Acupuncture has been reported to have significant effects, not only in alleviating impaired motor function, but also rescuing dopaminergic neuron deficits in rodent models of Parkinson's disease (PD). However, a systemic analysis of these beneficial effects has yet to be performed.

**Objective:** To evaluate the neuroprotective effect of acupuncture in animal models of PD.

**Methods:** A literature search of the PubMed, MEDLINE, EMBASE, China National Knowledge Infrastructure, Research Information Service System, and Japan Society of Acupuncture and Moxibustion databases was performed to retrieve studies that investigated the effects of acupuncture on PD. The quality of each included study was evaluated using the 10-item checklist modified from the Collaborative Approach to Meta-Analysis and Review of Animal Data from Experimental Studies. RevMan version 5.3 (Foundation for Statistical Computing, Vienna, Austria) was used for meta-analysis.

**Results:** The 42 studies included scored between 2 and 7 points, with a mean score of 4.6. Outcome measures included tyrosine hydroxylase (TH) level and dopamine content. Meta-analysis results revealed statistically significant effects of acupuncture for increasing both TH levels (33.97 [95% CI 33.15–34.79]; *p* < 0.00001) and dopamine content (4.23 [95% CI 3.53–4.92]; *p* < 0.00001) compared with that observed in PD control groups. In addition, motor dysfunctions exhibited by model PD animals were also mitigated by acupuncture treatment.

**Conclusions:** Although there were limitations in the number and quality of the included studies, results of this analysis suggest that acupuncture exerts a protective effect on dopaminergic neurons in rodent models of PD.

## Introduction

Parkinson's disease (PD) is a neurodegenerative disorder first described by Dr. James Parkinson in 1817 as a “shaking palsy” (Demaagd and Philip, 2015). PD is characterized by motor symptoms, such as rigidity, resting tremors, and postural instability, and non-motor symptoms including sleep disturbance, hallucinations, and constipation (Demaagd and Philip, 2015). It has been reported that 1–2% of the global population >65 years of age is affected by PD (Alves et al., 2008). In terms of pathology, recent studies have suggested that PD is closely associated with the loss of dopaminergic (DA) neurons in the substantia nigra (SN) pars compacta of the brain caused by familial and/or sporadic factors (Zhou et al., 2008; Surmeier et al., 2010; Blesa et al., 2015). Levodopa has been widely used in recent decades for the management of PD; however, complications following the use of levodopa are considerable.

In East Asian countries, acupuncture has long been used to treat motor dysfunction and brain disorders such as PD (Joh et al., 2010). Moreover, in recent years, it has been shown that acupuncture improves motor function in rodent models of PD via mechanisms including anti-inflammatory and neurotrophic effects (Yu et al., 2010; Rui et al., 2013). Furthermore, the effect of acupuncture on PD has been demonstrated in clinical studies. Improvement in motor function in PD patients who underwent bee venom acupuncture treatment has been reported (Cho et al., 2012), and motor function-associated neural responses with acupuncture have also been shown in PD patients using functional magnetic resonance imaging fMRI (Chae et al., 2009; Yeo et al., 2012). Additionally, a systematic review of clinical studies of acupuncture involving PD patients demonstrated the potential effectiveness of acupuncture (Lam et al., 2008). Accordingly, acupuncture has been suggested as an integrative medicine treatment for PD.

As mentioned earlier, destruction and recovery of DA neurons in the SN, which play critical role in motor functions, is significant in terms of the pathology of PD. Thus, it is important to investigate the extent to which acupuncture treatment affects the recovery of DA neurons. Rodent models have been widely used in PD research because they can provide valuable information in terms of understanding pathogenic processes and developing effective therapies (Duty and Jenner, 2011; Blandini and Armentero, 2012).

Recent rodent-based studies have demonstrated that acupuncture recovered DA neurons in a mouse model of 1-methyl-4-phenyl-1,2,3,6-tetrahydropyridine (MPTP)-induced PD, and in a 6-hydroxydopamine (6-OHDA)-induced PD rat model (Kim et al., 2005; Jeon et al., 2008; Park et al., 2015). In contrast, it was also reported that acupuncture treatment did not demonstrate a neuroprotective effect in an MPTP mouse model (Yang et al., 2017).

Therefore, the present systematic review and meta-analysis aimed to assess the pre-clinical evidence supporting the neuroprotective effects of acupuncture in rodent models of PD.

## Methods

### Literature Search

English-language studies that examined the neuroprotective effect of acupuncture in PD rodent models were included in the present study. The PubMed, EMBASE, and MEDLINE, China National Knowledge Infrastructure, Research Information Service System, and Japan Society of Acupuncture and Moxibustion databases were searched from inception until June 2018 using the following search terms: “mouse (mice)” or “rat (rats),” “acupuncture (electroacupuncture),” and “Parkinson's disease.”

### Inclusion/Exclusion Criteria

Studies were included based on the following criteria: subjects (rodent models of PD); intervention (acupuncture as the main intervention, but limited to manual acupuncture [MA] and electroacupuncture [EA]); and outcomes (tyrosine hydroxylase [TH] and DA neuron level were the main outcomes to evaluate the efficacy of acupuncture). Behavioral test data were the subsequent outcome to evaluate motor functions in PD rodent models. Studies not reporting exact outcome values and full-text articles not published in English were excluded.

### Data Extraction

Two authors (Kim and Ko) extracted the data independently. Data extracted from the databases included the following: publication year, name of the first author, and type of rodent PD model; type of acupuncture; results of behavioral tests; and the outcome of treatment in acupuncture-treated groups. Three studies were excluded because exact outcome values were not reported; thus, 42 original research articles were selected for further analysis.

### Quality Assessment

The methodological quality of each included study was assessed by two authors (Kim and Ko) using a 10-item checklist modified from the Collaborative Approach to Meta-Analysis and Review of Animal Data from Experimental Studies checklist (Sena et al., 2007): publication in a peer-reviewed journal; statements describing temperature control; random allocation to treatment or control; blinded building of the model; use of aged animal models; blinded assessment of outcome; use of anesthetic without significant intrinsic neuroprotective activity; sample size calculation; compliance with animal welfare regulations; and declarations of any potential conflicts of interest. A sum of the quality scores was recorded for each article, with a possible total score of 10 points.

### Statistical Analysis

In each study, densitometry of TH-positive (TH+) staining or stereological cell counting results or dopamine content were considered as continuous data. Because the same comparison was used in the studies (i.e., compared with a control group), the mean differences for effect sizes were estimated based on a fixed-effects model. Publication bias was assessed using a funnel plot. To examine the influence of the type of rodent model on the outcome measures, specific subgroups were defined: MPTP-induced PD model; 6-OHDA-induced PD model; medial forebrain bundle (MFB)-axotomy-induced PD model; and an alpha-synuclein (α-syn) mutation PD model.

The meta-analysis was performed using RevMan version 5.3 (Foundation for Statistical Computing, Vienna, Austria). The confidence interval was established at 95%, and *p* < 0.05 was considered to be statistically significant. For the assessment of study heterogeneity, the chi-squared distribution and I^2^ statistic were used.

## Results

### Study Inclusion

**Figure 1:**
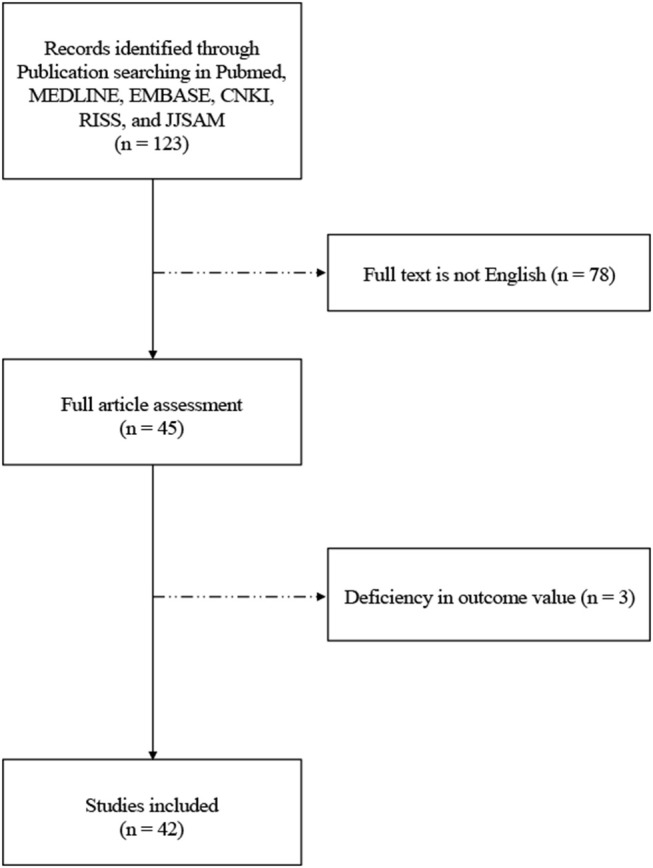
Flow diagram of study selection process.

Among 123 initially identified studies, 78 were excluded because the full-texts were not available in English. Full-text screening was performed for the remaining 45 studies, of which 3 were excluded due to deficiency in exact outcome values. A total of 42 studies were, therefore, included in the present review. A flow diagram of the study selection process is shown in Figure 1.

### Study Characteristics

Of the 42 studies, 23 used EA and 19 used MA. Of the PD models used in these studies, 21 used an MPTP-induced PD model, 15 used a 6-OHDA-induced PD model; and 5 other studies used an MFB lesion-induced PD model. One study used an A53T α-syn transgenic mouse model.

### Quality Assessment

The quality assessment of the included studies is summarized in Table 1. The quality score of the included studies ranged from 2 to 7 of a total 10 points. One study scored 7, 11 scored 6, 13 scored 5, 8 scored 4, 6 scored 3, and 3 studies scored 2 points. All the 42 studies were peer-reviewed and included randomly allocated control and acupuncture groups. Fifteen studies included statements describing temperature control, 17 described blinded assessment of outcomes, and 25 reported use of anesthetic without significant intrinsic neuroprotective activity. Thirty-four studies reported compliance with animal welfare regulations and 20 declared potential conflicts of interest. No study conducted blind building of the model or sample size calculation. Finally, no study used aged animals.

**Table 1:** Quality assessment.

**References**	**Q1**	**Q2**	**Q3**	**Q4**	**Q5**	**Q6**	**Q7**	**Q8**	**Q9**	**Q10**	**Score**
Liang et al., 2002	✓		✓				✓				3
Liang et al., 2003	✓		✓				✓		✓		4
Park et al., 2003	✓	✓	✓			✓	✓		✓		6
Kim et al., 2005	✓	✓	✓				✓				4
Kang et al., 2007	✓		✓				✓				3
Jeon et al., 2008	✓		✓				✓		✓	✓	5
Choi et al., 2009	✓		✓				✓				3
Jia et al., 2009	✓		✓			✓	✓				4
Hong et al., 2010	✓	✓	✓						✓		4
Jia et al., 2010	✓		✓						✓		3
Kim et al., 2010	✓		✓						✓		3
Yu et al., 2010	✓	✓	✓			✓	✓		✓		6
Choi et al., 2011a	✓		✓				✓		✓		4
Choi et al., 2011b	✓		✓				✓		✓		4
Kim et al., 2011a	✓		✓			✓			✓	✓	5
Kim et al., 2011b	✓		✓			✓			✓	✓	5
Wang et al., 2011	✓	✓	✓			✓			✓	✓	6
Yeo et al., 2013	✓		✓				✓		✓	✓	5
Huo et al., 2012	✓		✓			✓	✓		✓	✓	6
Sun et al., 2012	✓		✓			✓	✓		✓		5
Rui et al., 2013	✓	✓	✓			✓	✓		✓		6
Wang et al., 2013	✓	✓	✓			✓			✓		5
Yu et al., 2016	✓		✓			✓	✓		✓		5
Kim et al., 2014	✓		✓			✓	✓		✓		5
Deng et al., 2015	✓		✓				✓		✓	✓	5
Lv et al., 2015	✓		✓			✓	✓		✓	✓	6
Park et al., 2015	✓	✓	✓						✓	✓	5
Shen et al., 2015	✓		✓						✓	✓	4
Yeo et al., 2015	✓		✓						✓		3
Jia et al., 2017	✓		✓			✓	✓		✓	✓	6
Sun et al., 2016	✓	✓	✓			✓	✓		✓	✓	7
Tian et al., 2016	✓		✓								2
Yang et al., 2011	✓	✓	✓				✓		✓	✓	6
Yang et al., 2017	✓	✓	✓			✓	✓		✓	✓	6
Park et al., 2017	✓	✓	✓			✓			✓	✓	6
Jeon et al., 2017	✓	✓	✓						✓	✓	5
Lee et al., 2018	✓	✓	✓						✓	✓	5
Lu et al., 2017	✓	✓	✓				✓		✓	✓	6
Li et al., 2017a	✓		✓								2
Li et al., 2017b	✓		✓								2
Lin et al., 2017	✓		✓						✓	✓	4
Wang et al., 2018	✓		✓				✓		✓	✓	5

*Q1, publication in a peer-reviewed journal; Q2, statements describing control of temperature; Q3, random allocation to treatment or control; Q4, blinded building of model; Q5, use of aged animal model; Q6, blinded assessment of outcome; Q7, use of anesthetic without significant intrinsic neuroprotective activity; Q8, sample size calculation; Q9, compliance with animal welfare regulations; Q10, declared any potential conflict of interest*.

### Effect of Acupuncture on DA Neuron Protection

**Figure 2:**
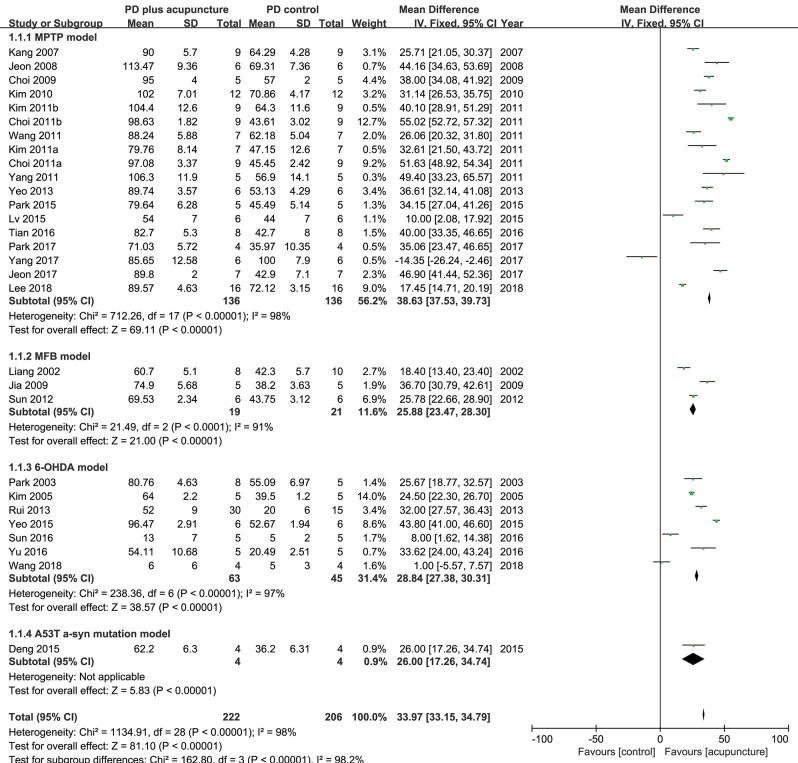
Comparison of tyrosine hydroxylase-positive neurons in substantia nigra with PD animal and acupuncture-treated PD animal.

**Figure 3:**
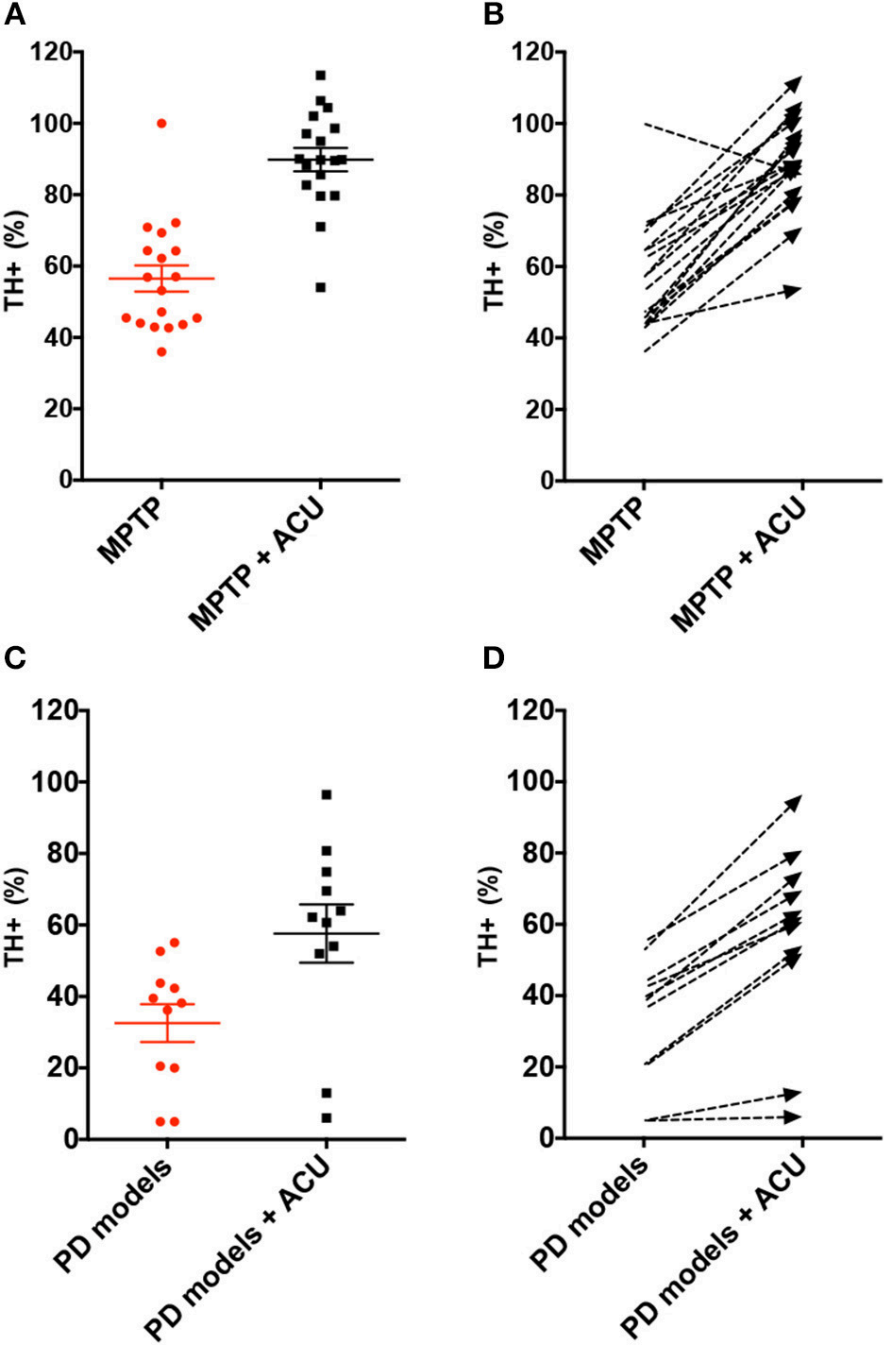
Analysis of difference in TH-positive level between PD rodent and acupuncture treatment group throughout studies. **(A)** Each TH+ level (% control) of MPTP-induced PD (MPTP) group and MPTP plus acupuncture (MPTP + ACU) group. Lines indicates the mean value and error. **(B)** Dashed arrows mean the slope of difference from MPTP to MPTP plus acupuncture group in each study. **(C)** Each TH+ level (% control) of 6-OHDA or MFB-axotomy or a-syn mutation-induced PD (PD models) group and acupuncture-treated PD (PD models + ACU) group. Lines indicates the mean value and error. **(D)** Dashed arrows mean the slope of difference between PD models to PD models plus acupuncture group in each study. Notice the difference of the TH+ level between acupuncture treated group compared to PD rodent model.

TH has a specific role in dopamine synthesis and is abundantly expressed in DA neurons; accordingly, it has been used as a dopamine neuronal marker in PD studies. Figure 2 shows the meta-analysis of studies with TH+ neurons in the SN of PD model animals. Twenty-nine studies adopted TH+ level as an outcome index. All of the studies reported a positive effect of acupuncture on increasing TH+ levels in the SN of acupuncture treated PD models compared with the PD control group except for two studies (Yang et al., 2017; Wang et al., 2018) (*n* = 426; standardized mean difference [SMD] 33.97; 95% CI 33.15–34.79; *p* < 0.00001; heterogeneity χ^2^ = 162.80, *I*^2^ = 98.2%, Figure 2). Each data point on the plot is shown with group comparisons (Figure 3). One study reported decreased TH+ cells in the acupuncture-treated group compared with the MPTP model (Figures 2, 3A,B), and one study demonstrated no difference in TH+ cells between acupuncture and 6-OHDA mouse models (Figures 2, 3C,D). However, TH+ cells were increased after acupuncture in almost all studies, except two that reported no significant difference (Figures 2, 3). Overall, integrated changes of TH+ neurons in PD models demonstrated 35.94% of normal brain and, interestingly, those of acupuncture treated improved these neuronal deficits by 70.43% (Figure 3). Additionally, a subgroup analysis of different PD models was performed to examine the effect of acupuncture on TH+ level. The result of the subgroup analysis indicated that acupuncture had a significant effect on MPTP models (SMD 56.2 [95% CI 37.53–39.73]; *p* < 0.00001), MFB models (SMD 25.88 [95% CI 23.47–28.30]; *p* < 0.00001), and 6-OHDA models (SMD 28.84 [95% CI 27.38–30.31]; *p* < 0.00001).

### Effect of Acupuncture on Dopamine Content Alteration

**Figure 4:**
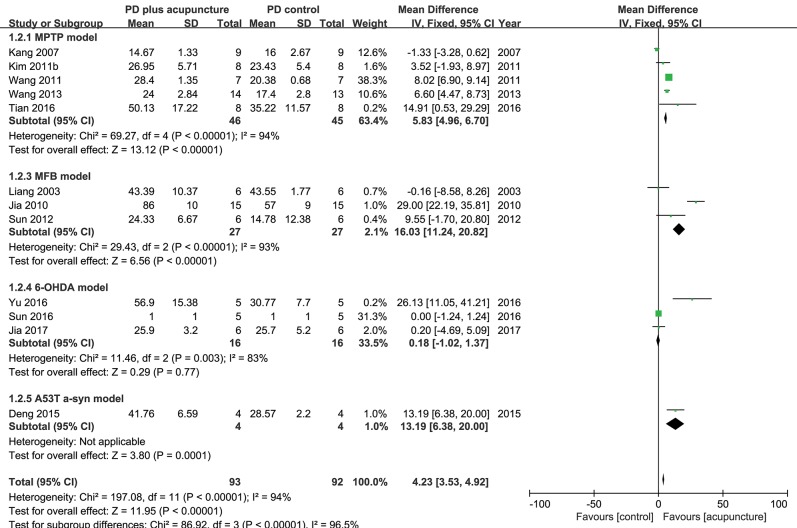
Comparison of dopamine content in striatum with PD animal and acupuncture-treated PD animal.

**Figure 5:**
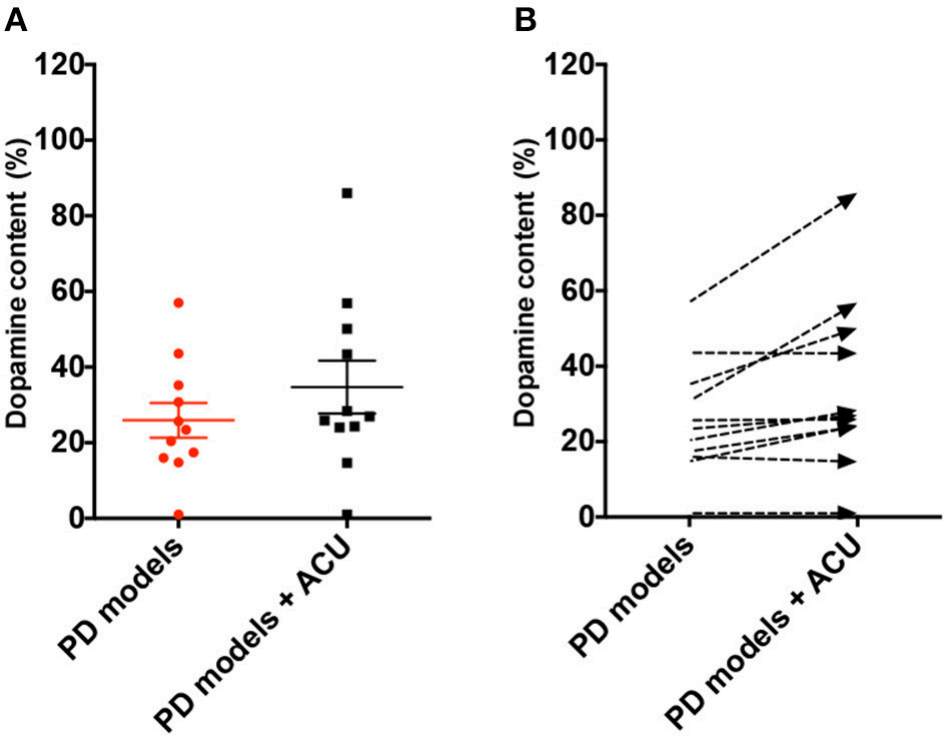
Analysis of difference in dopamine content level between PD rodent and acupuncture treatment group throughout studies. **(A)** Each dopamine content level (% control) of all the PD animal models (PD models) group and acupuncture-treated PD animal models (PD models + ACU) group. Lines indicates the mean value and error. **(B)** Dashed arrows mean the slope of difference from PD models to PD models plus acupuncture group in each study.

DA neuronal deficit leads to decreases in dopamine content in striatal projections. The above analysis indicated that DA neurons in PD model rodents were recovered by acupuncture; thus, it was explored how dopamine content was changed by acupuncture in the studies (Figure 4). Twelve studies reported the effect of acupuncture on improving dopamine content in PD models compared with the control PD group. There was no remarkable increase in dopamine content, except in three studies with high increase (Jia et al., 2010; Tian et al., 2016; Yu et al., 2016) (*n* = 185; SMD 4.23 [95% CI 3.53–4.92]; *p* < 0.00001; heterogeneity χ^2^ = 86.92, *I*^2^ = 96.5%, Figure 4). While three studies reported large increases in the striatal dopamine by acupuncture, overall, studies showed that dopamine content was not significantly altered by acupuncture (Figures 4, 5). In the subgroup analysis of dopamine content, there was a significant effect of acupuncture was observed in MPTP models (SMD 5.83 [95% CI 4.96–6.70]; *p* < 0.00001), MFB models (SMD 16.03 [95% CI 11.24–20.82]; *p* < 0.00001), but no significant difference was found in the 6-OHDA models (SMD 0.18 [95% CI −1.02–1.37]; *p* = 0.77).

### Effect of Acupuncture on Motor Function in a PD Model

Table 2 shows how acupuncture affected motor function in PD models by examining the results of behavioral tests. Among the 42 studies, 13 conducted a rotarod test, 3 conducted a pole test, 5 performed the open field test, 11 examined rotational behavior, and the 5 remaining studies performed the Morris Water Maze test, grip strength test, gait analysis, cylinder test, and locomotor test, respectively. For studies that used the rotarod test, it was evident that motor dysfunctions were alleviated in the treatment group compared with the PD rodent model group. For the pole test result, descending time was shortened in the acupuncture-treated group in three studies. In studies that examined rotation behavior between model group and treatment group, acupuncture reduced rotational asymmetry induced by MFB-axotomy or on the side of neurotoxin injection. Based on most studies, it is believed that acupuncture alleviated motor dysfunction in PD rodent models.

**Table 2:** Behavior tests and changes by acupuncture treatment in PD animal studies.

**References**	**Result**	**Behavior tests**
Liang et al., 2002	N/A	
Liang et al., 2003	Improved	Rotational behavior ↓
Park et al., 2003	Improved	Rotational behavior ↓
Kim et al., 2005	Improved	Rotational behavior ↓
Kang et al., 2007	N/A	
Jeon et al., 2008	Improved	Pole test ↓
Choi et al., 2009	N/A	
Jia et al., 2009	Improved	Rotational behavior ↓
Hong et al., 2010	N/A	
Jia et al., 2010	Improved	Rotarod ↑
Kim et al., 2010	N/A	
Yu et al., 2010	Improved	Rotational behavior ↓
Choi et al., 2011a	N/A	
Choi et al., 2011b	N/A	
Kim et al., 2011a	Improved	Rotarod ↑
Kim et al., 2011b	Improved	Rotarod ↑
Wang et al., 2011	N/A	
Yang et al., 2011	Improved	Pole test ↓
Huo et al., 2012	Improved	Rotational behavior ↓
Sun et al., 2012	Improved	Rotarod ↑
Rui et al., 2013	Improved	Rotational behavior ↓
Wang et al., 2013	Improved	Open field ↑
Yeo et al., 2013	N/A	
Kim et al., 2014	N/A	
Deng et al., 2015	Improved	Rotarod ↑ Open field ↑ Grip strength ↑ Gait analysis ↑
Lv et al., 2015	Improved	Open field ↑ (vertical activity)
Park et al., 2015	Improved	Rotarod ↑ Cylinder test ↑
Shen et al., 2015	N/A	
Yeo et al., 2015	N/A	
Jia et al., 2017	Improved	Rotarod ↑
Sun et al., 2016	Improved	Rotarod ↑ Rotational behavior ↓ Open field ↑
Tian et al., 2016	Improved	Rotarod ↑
Yu et al., 2016	Improved	Rotarod ↑ Cylinder test ↑ Locomotion **↑**
Yang et al., 2017	N/A	
Park et al., 2017	Improved	Rotarod ↑
Jeon et al., 2017	Improved	Pole test ↓
Lee et al., 2018	Improved	Pole test ↓
Lu et al., 2017	Improved	Morris water maze ↓
Li et al., 2017a	N/A	
Li et al., 2017b	Improved	Rotarod ↑ Open field ↑
Lin et al., 2017	Improved	Rotational behavior ↓ Locomotion **↑**
Wang et al., 2018	Improved	Rotarod ↑ Rotational behavior ↓

*Improved, motor dysfunction by PD was mitigated by acupuncture; N/A, not applicable; ↑, increase of the value compared to PD model; ↓, decrease of the value compared to PD model*.

## Discussion

### Summary of Evidence

In this review, we systematically analyzed 42 acupuncture studies that used rodent models of PD to determine whether acupuncture can improve PD symptoms and/or pathology. It is possible to study the progression of PD and therapeutic approaches by using rodent models. However, there is still no experimental rodent model that can perfectly phenocopy the disease (Jagmag et al., 2015). There was a broad range of experimental models used to study PD including MPTP mouse, 6-OHDA-lesioned rat, MFB-axotomy rodent, and an α-syn mutation mouse. In the studies, acupuncture treatment involved MA and EA. Overall, meta-analysis revealed that deficits of both TH+ levels and dopamine content in PD model animals were recovered by acupuncture. Acupuncture treatment in MPTP, MFB, and 6-OHDA models were also found to be effective according to subgroup analyses. Additionally, motor dysfunctions in those PD models were also alleviated by acupuncture.

### Possible Mechanism of Neuroprotective Effects

Several mechanisms of acupuncture have been suggested to be involved in recovering DA neuronal deficits. First, the neurotrophic factor-induced cell proliferation pathway was suggested as a potential mechanism to explain the neuroprotective effect of acupuncture. For example, it was found that acupuncture increased brain-derived neurotrophic factor (BDNF) levels, followed by activation of TrkB-related cell proliferation cascade (Liang et al., 2002; Park et al., 2003; Sun et al., 2016). Glial cell-derived neurotrophic factor was also upregulated by acupuncture (Liang et al., 2003), and there was remarkable increase in cyclophilin A levels (Jeon et al., 2008). Additionally, it was found that acupuncture activates hypothalamic melanin-concentrating hormone (MCH), which is involved in neuronal protection by upregulating a downstream pathway related to neuroprotection in the SN of MPTP-induced and A53T α-syn mutant PD mice (Park et al., 2017). Moreover, various researchers have suggested possibilities that acupuncture helps PD patients recover from PD through biological processes such as anti-oxidant (Yu et al., 2010; Wang et al., 2011; Lv et al., 2015; Lee et al., 2018), anti-inflammation (Kang et al., 2007; Jeon et al., 2008; Deng et al., 2015), and regulation of autophagy (Tian et al., 2016). Although all of these processes need to be clinically verified, the scientific evidence revealed strong possibilities of a neuroprotective effect of acupuncture on PD.

### Contradictory Results and Plausible Reasons

In contrast, we found that one study reported non-meaningful changes in TH+ levels (Jia et al., 2017) and contradicting results in another study (Yang et al., 2017), which reported that acupuncture is not effective in improving DA neuron protection. In a study by Yang et al., the authors reported that the number of TH+ neurons did not increase as a result of acupuncture treatment compared with PD models. However, this study did not have a normal control group and, as such, it is questionable whether a PD model was successfully established. Although C57/Bl6 mice are the most sensitive to MPTP, there are some cases in which models are not successfully induced (Jackson-Lewis and Przedborski, 2007). This illustrates why thorough controls, such as saline-injected normal mice, are needed for developing reliable rodent models. Therefore, it is doubtful whether their model would be appropriate to examine the effects of acupuncture in PD rodent models. In a study by Jia et al., changes in TH+ neurons were not meaningful but were partially restored. The study used 6-OHDA surgery-induced neuronal depletion model for development of PD. We found that the degree of neuronal deficit by 6-OHDA surgery was worse than neurotoxin injection (28.25 vs. 56.52% normal control TH+ value of 6-OHDA and MPTP, respectively) (Figure 5). Such differences among models may also lower the degree of recovery effect of acupuncture (52.33 vs. 89.83% normal control TH+ value of 6-OHDA+ACU and MPTP+ACU, respectively) (Figure 5). It also explains why the recent development of PD animal models remains arguable (Duty and Jenner, 2011; Blesa et al., 2012); therefore, it is important to carefully examine studies using various models to induce PD to draw the appropriate conclusions.

### Dopamine Content and Possible Mechanisms for Improvements in Motor Function

Studies have shown that dopamine content is recovered, although not as much as neuronal deficits, by acupuncture treatment (Figures 4, 5). These results were unexpected because most studies reported that motor dysfunctions in PD animals were significantly altered after acupuncture (Table 2). Studies have reported that partial recovery of DA neurons is not sufficient to rescue neuronal function completely in terms of dopamine secretion. Instead, studies have suggested that acupuncture treatment has another effect on PD animals, which is improvement of synaptic function in addition to neuronal protection (Jia et al., 2009; Kim et al., 2011b; Yang et al., 2011; Sun et al., 2012; Rui et al., 2013; Yu et al., 2016). Notably, single-photon emission computed tomography imaging has demonstrated that neurotransmission was increased by acupuncture (Yang et al., 2011). Moreover, analysis of synaptic changes showed that dopamine use was enhanced via regulating the D1 dopamine receptor and dopamine transporter (Rui et al., 2013). Additionally, *in vivo* microdialysis results have shown that dopamine availability (Kim et al., 2011b) and other neurotransmitters, such as acetylcholine and glutamate, were mitigated by acupuncture (Sun et al., 2012). Furthermore, postsynaptic cortico-striatal pathway alteration was found after acupuncture (Wang et al., 2018). Based on these results, it is possible to hypothesize that acupuncture can modulate DA synaptic pathways via alteration of neuronal plasticity; however, it rescues less striatal dopamine content itself in PD animal models.

### Limitations and Future Direction

There were several limitations in terms of drawing definitive conclusions based on the included studies. First, it is difficult to perform an accurate analysis by including studies using a variety of PD models and acupuncture methods. Nevertheless, it is meaningful that the potential neuroprotective effect of acupuncture treatment was evident in most of the included studies. Second, the number of available studies was not conducive to a thorough systematic review, which reflects the limited research investigating effects of acupuncture. In other words, more studies will enable us to draw more accurate conclusions in the future.

## Conclusion

Results of the present review and analysis suggest that acupuncture treatment potentially protected DA neurons through various beneficial mechanisms. Nevertheless, resolving the low quality of studies and further research investigating the efficacy of different acupuncture treatment methods in PD rodent models will be needed.

## Author Contributions

JK analyzed the data. S-NK and H-JP supervised the project. JK, HL, S-NK, and H-JP wrote the paper.

### Conflict of Interest Statement

The authors declare that the research was conducted in the absence of any commercial or financial relationships that could be construed as a potential conflict of interest.
